# SVM-Enabled Intelligent Genetic Algorithmic Model for Realizing Efficient Universal Feature Selection in Breast Cyst Image Acquired via Ultrasound Sensing Systems

**DOI:** 10.3390/s20020432

**Published:** 2020-01-12

**Authors:** Chuan-Yu Chang, Kathiravan Srinivasan, Mao-Cheng Chen, Shao-Jer Chen

**Affiliations:** 1 Department of Computer Science and Information Engineering, National Yunlin University of Science and Technology, Yunlin 64002, Taiwan; winersbaby@gmail.com; 2 School of Information Technology and Engineering, Vellore Institute of Technology (VIT), Vellore 632014, India; kathiravan.srinivasan@vit.ac.in; 3 Department of Medical Imaging, Buddhist Dalin Tzu Chi General Hospital, Chiayi 622, Taiwan; shaojer.chen@msa.hinet.net

**Keywords:** Sorensen-Dice coefficient, *t*-test, Pearson’s R measure, ultrasound sensing systems, SVM-enabled intelligent genetic algorithmic model, breast cyst imagery

## Abstract

In recent years, there are several cost-effective intelligent sensing systems such as ultrasound imaging systems for visualizing the internal body structures of the body. Further, such intelligent sensing systems such as ultrasound systems have been deployed by medical doctors around the globe for efficient detection of several diseases and disorders in the human body. Even though the ultrasound sensing system is a useful tool for obtaining the imagery of various body parts, there is always a possibility of inconsistencies in these images due to the variation in the settings of the system parameters. Therefore, in order to overcome such issues, this research devises an SVM-enabled intelligent genetic algorithmic model for choosing the universal features with four distinct settings of the parameters. Subsequently, the distinguishing characteristics of these features are assessed utilizing the Sorensen-Dice coefficient, *t*-test, and Pearson’s R measure. It is apparent from the results of the SVM-enabled intelligent genetic algorithmic model that this approach aids in the effectual selection of universal features for the breast cyst images. In addition, this approach also accomplishes superior accuracy in the classification of the ultrasound image for four distinct settings of the parameters.

## 1. Introduction 

In general, medical practitioners classify the breast cysts in two major categories, namely the benign and malignant tumors. It has to be noted that, even though the cysts observed in the breasts are technically stated as tumors, it can be witnessed that every observed breast tumor is not malignant (cancerous cell/tissue). Further, medical doctors state that eighty percent of the biopsied breast cysts are observed to be benign (noncancerous cell/tissue). Even though such breast cysts are benign in nature, it has to be removed surgically to avert its interference with the normal functioning of the breasts. Moreover, it can be seen that these benign breasts do not affect the neighboring cell/tissue, and it is not dangerous or life-threatening. On the other side, the malignant breast tumors are cancerous, and if it is not diagnosed and treated premature, it will cause severe damage to the neighboring cells and tissues. In addition, it gets spread to the nearby lymph glands (nodes). Further, these malignant cancerous cells start spreading throughout the body via the lymph system and bloodstream, which is known as metastasis. Moreover, breast cancer at this point is dangerous, and the probability of cure is very minimal. 

According to the study conducted by the Taiwanese government in 2015 and 2016, there was a substantial increase in deaths due to cancer [1]. In addition, this information is evident from the details portrayed in Table 1 [1]. Moreover, in Taiwan, breast cancer in females ranks fourth amongst the top 10 leading causes of cancer deaths for the year 2016. Therefore, many researchers are trying to devise better approaches for the early detection of breast cancer [2,3]. Extensive use of intelligent sensing in medical imaging has begun to correctly extract and visualize all the essential details of the internal human body structures in the field of medical diagnosis [4,5,6,7,8,9]. Further, various intelligent sensing modalities such as ultrasound sensing system, computerized axial tomography (CAT), positron emission tomography (PET), and nuclear magnetic resonance imaging (NMR) are helping medical doctors significantly in diagnosing several ailments and health disorders. Each intelligent sensing modalities mentioned above have their own properties and utilities. The diagnosis of cancers in the breast can be achieved through mammography (gold standard), which utilizes ionizing radiation (X-rays). Since these ionizing radiations are harmful in nature and can also enhance the growth of lesion cells, usage of nonionizing diagnosis methods such as ultrasound systems has become widely popular. Moreover, it has to be noted that women with dense breasts are easily prone to breast cancer. In addition, mammography exhibits low-sensitivity in detecting dense breasts with cancer. Therefore, the role of ultrasound sensing systems becomes significant in the effective diagnosis of dense breasts with cancer.

Furthermore, persistent exposure to such radiation might even be more dangerous for the subject under scanning. Moreover, the ultrasound sensing system is considered to be a safe option by medical physicians, as it uses sound waves and has no damaging radiation effects. Moreover, such ultrasound sensing systems are widely prevalent these days in the real-time diagnosis of diseases and health disorders.

In recent investigations, it has been found that when breast cancer is detected in earlier stages, it can be diagnosed and operated upon to remove cancer altogether from the patient [4,5,6,7,8,9]. Moreover, several physicians have recommended the deployment of sonography sensing systems for diagnosing cancer in the breasts. Even though the sonography sensing systems are well-renowned in the premature diagnosis of cancer in the breasts, on the other hand, it is a critical fact that the sonography sensing systems in some cases produce imagery with low-resolution; therefore, suitable approaches are essential for enhancing the resolution of these images. The imaging by the sonography sensing systems can only point out at suspected masses (cysts), and the biopsy is mainly used to assess if the sampled tissue is malignant or normal. In addition, the wastage of overall resources can be avoided by the use of a suitable methodology that will enable medical doctors to make an efficient detection of breast cancer during their primary examination of the imagery. The genetic algorithm (GA) is a well-known approach for solving pattern recognition problems in medical and random image datasets [10,11,12].

The works presented in [13] established a conventional genetic algorithm for feature selection; besides, they had deployed three distinct classifiers (neural networks, discriminant analysis, logistic regression) for classifying microcalcification patterns present in the digital mammograms. Moreover, they had implemented the digital database for screening mammography (DDSM) dataset obtained from the University of South Florida (http://www.eng.usf.edu/cvprg/Mammography/Database.html). The authors in [14] established a conventional genetic algorithm-based approach for feature subset selection and also for fixing the parameters involved in the SVM Classification. Further, they had applied this model to 11 different datasets (German-credit card, Australian-credit card; Pima-Indian diabetes, heart disease-Statlog project, breast cancer-Wisconsin, contraceptive method choice, Ionosphere, Iris, Sonar, Statlog project: Vehicle, Vowel) obtained from UCI repository (https://archive.ics.uci.edu/mL/datasets.php). The works [13] and [14] deployed the conventional genetic algorithm-based model with restricted (same) parameter settings. Moreover, these approaches were not validated under multiple distinct settings of the parameters, which makes them inappropriate for processing the ultrasound images of a patient obtained under various distinct settings of the parameters.

Further, it can be seen that numerous researchers have been working on developing better solutions for ultrasound sensing systems [15] and also for classifying the lesion regions [16,17]. Currently, a few deep-learning models have become popular in diagnosing the diseases from ultrasound image datasets [18]. The work in [18] briefly introduces several popular deep learning architectures, and then summarizes and thoroughly discusses their applications in various specific tasks in US image analysis, such as classification, detection, and segmentation. The authors in [19], discuss a Faster R-CNN based detector that is more suitable for thyroid papillary carcinoma detection in ultrasound images. The research in [20] establishes an application of deep learning for predicting the low-resolution map of fiber orientation in extracted muscle regions. However, the use of deep-learning models might be challenging when the ultrasound image dataset is a small one. The work in [21] developed a novel CAD system for mass detection and classification in breast ultrasound images based on the fuzzy SVM. However, their breast ultrasound image dataset was captured in a single (restricted) parameter setting, whereas the breast ultrasound image dataset in our work, has been captured with four different settings of the parameters. The work in [22] established a novel and accurate method based on ultrasound RF time series analysis and an extended version of support vector machine classification for generating probabilistic prostrate cancer maps, which can augment ultrasound images of the prostate and enhance the biopsy process. The work in [23] established an SVM system to characterize normal liver, cirrhotic liver, and hepatocellular carcinoma (HCC) using wavelet packet texture descriptors. Further, even though the works in [22] and [23] use the keyword SVM and ultrasound images, but it has to be noted that the work in [22] deploys a prostate ultrasound image dataset in a single (restricted) parameter setting and the work in [23] utilized the liver ultrasound images obtained in a single (restricted) parameter setting, whereas, our work is related to the breast ultrasound image dataset captured with four different settings of the parameters.

In general, three essential stages are part of the research methodology. The primary stage is generating the required ultrasound dataset imagery, which involves the segregation of the region-of-interest (ROI) for all the images. Then, the second stages include the extraction of the appropriate features using the segregated ROI imagery that is achieved by training the proposed model by utilizing the marked imagery. Subsequently, the third stage represents the classification of these ultrasound imageries. In addition, it is clearly evident that in several scenarios, validating the outcomes of the classification is quite challenging, since most of these approaches follow only a single (constrained) setting of the parameters. 

Usually, this scenario leads to a drastic decrease in the quality of the obtained outcomes. Since such a situation where the ultrasound sensing systems having distinct settings of the parameters, can produce diverse imagery for a single subject under study. Additionally, in the computer-aided diagnosis of breast cysts, several research teams have tried to segregate appropriate feature set for successful classification; still, there is a research gap in identifying suitable optimal feature sets for such a task. Therefore, the extraction of highly discriminative and unique features is an arduous and exciting task. Due to this fact, the proposed model is established, and it also generalizes (converges) for four distinct settings of the parameters.

The key contributions of this work are summarized as follows:

- This research devises an SVM-enabled intelligent genetic algorithmic model for choosing the universal features with four distinct settings of the parameters.
- In addition, this proposed approach categorizes the breast cyst imagery as breast mice (BM) and invasive ductal carcinoma (IDC).
- Moreover, the intelligent genetic algorithmic model amalgamates the benefits of wrapper algorithm and filter algorithm for choosing the highly discriminative and comprehensive set of features under four distinct settings of the parameters.
- The results of the proposed approach exhibit the fact that the chosen set of universal features enables superior classification outcomes under four distinct settings of the parameters.
- Furthermore, the proposed model also empowers the medical doctors in the detection of breast mice (BM) and invasive ductal carcinoma (IDC).

The further portions of this research are categorized as an illustration of the materials and methods available in Section 2, the outcomes of the experiments are elucidated in Section 3, and Section 4 establishes the conclusion of this research.

## 2. Materials and Methods

The ultrasound breast cyst imagery dataset was gathered from 23 different patients at the Department of Medical Imaging, Buddhist Dalin Tzu Chi General Hospital. The physician used four different settings of the parameter in GELOGIQ 700 for obtaining four diverse ultrasound breast images from each patient. Further, the physician outlined the ROI on each ultrasound breast image, and the system cut the ROI area into many 23 × 23 blocks. A total of 4038 samples with a size of 23 × 23 were collected, including 2012 samples of invasive ductal carcinoma and 2026 samples of breast mice. Among these samples, 50% were utilized for selecting the features and the rest for testing purposes. In this research, the SVM-enabled intelligent genetic algorithmic model is proposed for identifying a superior set of features under four distinct settings of the parameters in the ultrasound sensing systems. It has to be noted that this approach blends together the benefits of both the wrapper algorithm and the filter algorithm, respectively. In addition, during the process of accomplishing the filter algorithm, the features are assessed with the help of the metrics such as Sorensen-Dice coefficient; *t*-test; and Pearson’s R measure. Subsequently, the wrapper algorithm comprises the deployment of the intelligent genetic algorithmic approach for choosing the universal set of features that exhibits superior performance. 

**Figure 1:**
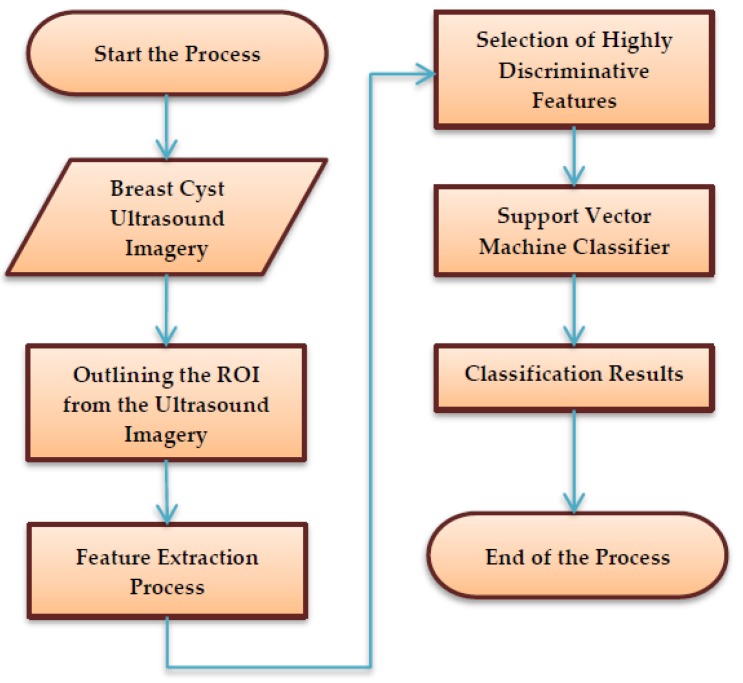
The schematic process flow diagram of the SVM (support vector machine)-enabled intelligent genetic algorithmic model.

The schematic process flow diagram of the SVM-enabled intelligent genetic algorithmic model is depicted in Figure 1. Primarily, the process begins with marking and segregating the ROI from the breast cyst ultrasound imagery by a medical practitioner based on his expertise, along with insights from the biopsy report. Then, it is followed by the extraction of 126 unique sets of features from the marked ROI ultrasound imagery utilizing a mask with a size of 23 × 23. Subsequently, the highly discriminative features that exhibit superior accuracy in classifying the images are chosen by deploying the intelligent genetic algorithmic approach. In the final step, the breast cyst ultrasound imagery is classified as breast mice and invasive ductal carcinoma with the assistance of a support vector machine (SVM) approach.

### 2.1. Outlining the Region-of-Interest from Ultrasound Image

**Figure 2:**
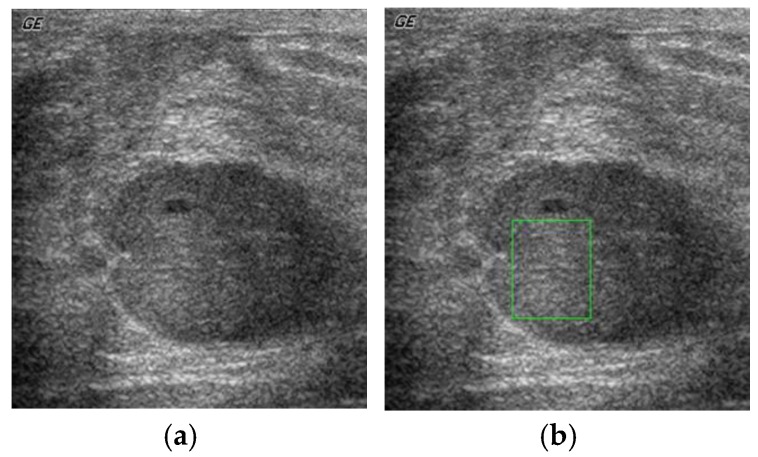
(**a**) General representation of the ultrasound breast cyst imagery, (**b**) marked and segregated ROI (region of interest) portions from this imagery.

The primary step is the feature set extraction from the ultrasound breast cyst imagery. Initially, the expert medical practitioner identifies, marks, and segregates the ROI from the ultrasound breast cyst image with the ideas taken from the biopsy report. In Figure 2a, we can observe the general representation of the ultrasound breast cyst image, and Figure 2b portrays the marked and segregated ROI portions from these imageries. 

### 2.2. Extraction of Feature Sets

In general, from all the ROI images, a mask with a size 23 × 23 is utilized for extracting the 126 feature sets. Further, all these feature sets can be exemplified as follows:

#### 2.2.1. Histogram Feature Sets

The statistical information from the ultrasound breast cyst imagery can be easily obtained utilizing the histogram feature sets. Further, the gray level distribution and related properties of the ultrasound breast cyst imagery can be computed employing the histogram. In addition, the histogram is utilized in this work to extract and compute seven sets of the feature that are represented in Table 2. 

#### 2.2.2. Gray-Level Spatial Dependence Matrix 

A statistical approach for assessing the texture that assumes the spatial association between the pixels is mentioned as the gray-level spatial dependence matrix (GLSDM) [24]. Further, this matrix produces distinct outcomes based on the distance and angle parameter values, respectively. It can be observed that the gray-level spatial dependence matrix aids in the computation of 13 sets of the feature represented in Table 3. 

#### 2.2.3. Statistical Feature Matrix

Generally, the statistical characteristics such as the distance between the pixels of the image is computed with the aid of the statistical feature matrix (SFM) [25]. In this research, we compute the dissimilarity (F21) feature using the statistical feature matrix, as shown in Table 4. 

#### 2.2.4. Gray Level Run-Length Textural Matrix 

The gray level run-length textural matrix can be computed using the image’s gray-level and the maximum amount of gray-level value that appears incessantly in a particular pathway [26]. The five sets of features can be computed utilizing the gray level run-length textural matrix (GLRLM), as depicted in Table 5.

#### 2.2.5. Laws’ Texture Energy Matrix

The core vector of the texture can be computed using the Law’s texture energy matrix [27]. The statistical outcomes are achieved by deploying the Law’s mask with the size 5 × 5 over the ultrasound breast cyst imagery. As a result, we could compute around ten sets of features utilizing this matrix, as represented in Table 6. 

#### 2.2.6. Neighboring Gray Level Dependence Matrix

Usually, the gray-level association between all the image pixels and its neighbors can be utilized for generating the neighboring gray level dependence matrix (NGLDM) [28]. The five sets of the features extracted using the neighboring gray level dependence matrix is represented in Table 7.

#### 2.2.7. Neighborhood Gray Tone Difference Matrix 

The neighborhood gray tone difference matrix (NGTDM) aids in attaining the spatial variation of the gray intensity via the notification of the gray tone difference amidst the pixel and its neighboring pixels in an image [29]. Then, neighborhood gray tone difference matrix is utilized for obtaining the five sets of the feature, as shown in Table 8. 

#### 2.2.8. Wavelet Transform Feature Sets

Typically, using the wavelet transform, the images can be disintegrated as the low–low frequency subband images utilizing the low pass filters [30]. In addition, the LL subband obtained using the wavelet transform is deployed for computing the forty-eight sets of features, as portrayed in Table 9.

#### 2.2.9. Fourier Features—Local Fourier Coefficients

The local Fourier coefficients of the ultrasound breast cyst imagery can be obtained by deploying the Fourier transform. Further, it can be observed that all the coefficients have two elements, namely, the magnitude and the phase angle. Moreover, utilizing the Fourier transform, we could compute around 32 features, as listed in Table 10.

### 2.3. SVM-Enabled Intelligent Genetic Algorithmic Model for Selecting Appropriate Features

In this work, the SVM-enabled intelligent genetic algorithmic model encompasses the selection of features portion that entails two stages; namely, the first stage is the filter algorithm stage, and the second one is the wrapper algorithm stage. Further, in the filter algorithm stage, the features possessing a low score are removed. In addition, this is accomplished through utilizing the Sorensen-Dice coefficient, *t*-test, and Pearson’s R measure. Then, the highly discriminative and superior subset of features is obtained by deploying the intelligent genetic algorithmic approach.

#### 2.3.1. Filter Algorithm

We observed that the previous section also dealt with the extraction of 126 feature sets. Furthermore, these sets of features become the input of the intelligent genetic algorithmic model. Moreover, another major point to be taken into account is that the set of features, which were obtained using four distinct settings of the parameters, become a diverse group. However, it has to be noted that the complexity of the overall computation becomes a challenging point at this juncture. Therefore, this arduous challenge can be surpassed through the deployment of the three assessment measures such as Sorensen-Dice coefficient, *t*-test, and Pearson’s R measure for scoring the extracted set of features and thereby eliminating the low-scoring and irrelevant features. The assessment metrics used in eliminating the irrelevant features are discussed beneath:

- Augmented Sorensen-Dice coefficient

The augmented version of the Sorensen-Dice coefficient of the *i*-th feature set for the ultrasound breast cyst datasets can be expressed using the following equation:

(1) $$F\left(i\right)=\frac{\sum_{j=1}^{s}{\left({\bar{x}}_{i}{}^{\left(j\right)}-{\bar{x}}_{i}\right)}^{2}}{\sum_{j=1}^{s}\frac{1}{n_{j}-1}\sum_{k=1}^{n_{j}}{\left({\bar{x}}_{k,i}^{\left(j\right)}-{\bar{x}}_{i}^{\left(j\right)}\right)}^{2}}$$

where the values ${\bar{x}}_{i}^{}$, and ${\bar{x}}_{i}^{\left(j\right)}$ signifies the average of the *i*-th feature in for all the ultrasound breast cyst datasets and the *j*-th ultrasound breast cyst dataset, correspondingly. Further, the value ${\bar{x}}_{k,i}^{\left(j\right)}$ specifies the average of the *i*-th feature of the *k*-th instance in the *j*-th ultrasound breast cyst dataset and s represents the total number of features.

- The *t*-test

The values of the *t*-test score for the *i*-th feature in the *j*-th dataset can be expressed using the following equation:

(2) $$T_{i}^{j}=\left|\frac{{\left({\bar{x}}_{i}^{j}{}^{\left(+\right)}-{\bar{x}}_{i}^{j}{}^{\left(-\right)}\right)}^{2}}{\sqrt{{\left(\sigma_{i}^{j}{}^{\left(+\right)}\right)}^{2}/n_{i}^{j}{}^{\left(+\right)}+{\left(\sigma_{i}^{j}{}^{\left(-\right)}\right)}^{2}/n_{i}^{j^{\left(-\right)}}}}\right|$$

where the values ${\bar{x}}_{i}^{j}{}^{\left(+\right)}$, and ${\bar{x}}_{i}^{j}{}^{\left(-\right)}$ specify the average value of the positive and negative instances of the *i*-th feature in the *j*-th ultrasound breast cyst dataset, correspondingly. The values $n_{i}^{j}{}^{\left(+\right)}$ and $n_{i}^{j}{}^{\left(-\right)}$ signify the total number of positive and negative instances of the *i*-th feature in the *j*-th ultrasound breast cyst dataset, correspondingly. Further, the values $\sigma_{i,j}{}^{\left(+\right)}$ and $\sigma_{i,j}{}^{\left(-\right)}$ characterize the standard deviation of the *i*-th feature of the positive and negative instances in the *j*-th ultrasound breast cyst dataset, correspondingly. The *t*-test score of the *i*-th feature in all ultrasound breast cyst datasets can be stated using the following equation:

(3) $$T_{i}=\frac{\sum_{j=1}^{s}T_{i}^{j}}{s\times \sigma \left(T_{i}^{1},T_{i}^{2},\ldots ,T_{i}^{s}\right)}$$

where s represents the total number of ultrasound breast cyst datasets.

- Pearson’s R measure

Pearson’s R measure of the *k*-th feature between ultrasound breast cyst datasets i and j can be expressed using the following equation:

(4) $$r_{k}^{i,j}=\frac{\sum_{p}\left(x_{k}^{i}-{\bar{x}}_{k}^{i}\right)\left(x_{k}^{j}-{\bar{x}}_{k}^{j}\right)}{\sqrt{\sum_{p}{\left(x_{k}^{i}-{\bar{x}}_{k}^{i}\right)}^{2}}\sqrt{\sum_{p}{\left(x_{k}^{j}-{\bar{x}}_{k}^{j}\right)}^{2}}}$$

where the factors $x_{k}^{i}$ and $x_{k}^{j}$ specify the value of the feature k in *i*-th and *j*-th ultrasound breast cyst dataset, correspondingly. Further, the factors ${\bar{x}}_{k}^{i}$ and ${\bar{x}}_{k}^{j}$ signify the mean values of $x_{k}^{i}$ and $x_{k}^{j}$ averaged over p occurrences. Moreover, after the computation of the Pearson’s R measure for all likely groupings of the ultrasound breast cyst datasets, the Pearson’s R measure of the *k-*th feature in each ultrasound breast cyst dataset is computed. 

The Pearson’s R measure of the feature k in ultrasound breast cyst dataset i can be expressed using the following equation:

(5) $$Cor_{k}^{i}=\frac{\sum_{j=1}^{s}\left|r_{k}^{i,j}\right|}{s-1}\;\mathrm{if}\;i\neq j$$

where s signifies the total number of ultrasound breast cyst dataset. Finally, Pearson’s R measure of the *k*-th feature for all ultrasound breast cyst datasets can be computed using the following equation:

(6) $$Cor_{k}=\frac{\sum_{i=1}^{s}Cor_{k,i}}{s\times \sigma \left(Cor_{k,1},Cor_{k,2},\ldots ,Cor_{k,s}\right)}$$

Moreover, after accomplishing the computation of the assessment metrics such as Sorensen-Dice coefficient, *t*-test, and Pearson’s R measure for all the sets of features, three scores for each set of features are listed. Subsequently, these features are sorted down in the decreasing order based on their assessment scores. Further, the set of irrelevant features with low assessment scores are removed. Consequently, the support vector machine classifier is utilized for computing the accuracy of classification for these set of features. Lastly, the set of ranked features in both these lists that intersect gets selected for the next phase, namely the wrapper algorithm.

#### 2.3.2. Wrapper Algorithm

Usually, selecting the features that yield superior accuracy in classifying the ultrasound breast cyst datasets for four distinct settings of the parameters is achieved through this stage. Moreover, the SVM-enabled intelligent genetic algorithm gets utilized as the wrapper. The intelligent genetic algorithm can be seen as a smart approach for searching the highly discriminant features, which impersonates the natural selection approach. Subsequently, based on the principle known as the “survival of the fittest,” this intelligent genetic algorithm achieves optimal outcomes after a sequence of iterations. It can be observed that in this algorithm, all the chromosomes are depicted by means of a bit or an integer string. Consequently, the intelligent genetic algorithm has three major phases, namely, the reproduction, the crossover, and the mutation. Therefore, in the reproduction phase, the chromosomes are selected based on the assessment of their fitness values. Then, the crossover and mutation phases are the critical stages for producing the next generation chromosomes. In addition, the fitness function, design of chromosomes, and the intelligent genetic algorithm are discussed in the subsequent subsection.

- Fitness Function

We have initially stated that the key objective of this research is determining the universal sets of the feature that achieves superior classification outcomes for four distinct settings of the parameters. Therefore, the accuracy of the classification and the variance in the accuracy for all four distinct settings of the parameters play a vital role in designing the fitness function. In addition, the following equation represents the fitness function, which obeys the principles as mentioned above:

(7) $$\begin{array}{l} \mathrm{fitness}=\sum_{i=1}^{s}Acc\_S_{i}-\\ \left[\begin{array}{l} {\left(Acc\_S_{1}-Acc\_S_{2}\right)}^{2}+{\left(Acc\_S_{1}-Acc\_S_{3}\right)}^{2}+\ldots \\ +{\left(Acc\_S_{1}-Acc\_S_{N}\right)}^{2}+{\left(Acc\_S_{2}-Acc\_S_{3}\right)}^{2}+\ldots \\ +{\left(Acc\_S_{2}-Acc\_S_{N}\right)}^{2}+\ldots +{\left(Acc\_S_{N-1}-Acc\_S_{N}\right)}^{2} \end{array}\right] \end{array}$$

where *s* specifies the total number of ultrasound breast cyst images, the value $Acc\_S_{{}_{i}}$ signifies the accuracy of classification for the *i*-th ultrasound breast cyst images.

- Design of Chromosomes

**Figure 3:**
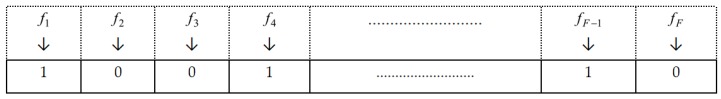
The schematic diagram of the binary encoding of the chromosome.

In the design of chromosomes, each chromosome can be portrayed utilizing an n-digit binary string. Further, the factor “F” signifies the total number of feature sets. Moreover, individual chromosomes designate either the digit “one” or “zero” to indicate the selection of a feature. The schematic diagram of the binary encoding of the chromosome is depicted in Figure 3.

- Feature Selection Using the Intelligent Genetic Algorithm

**Figure 4:**
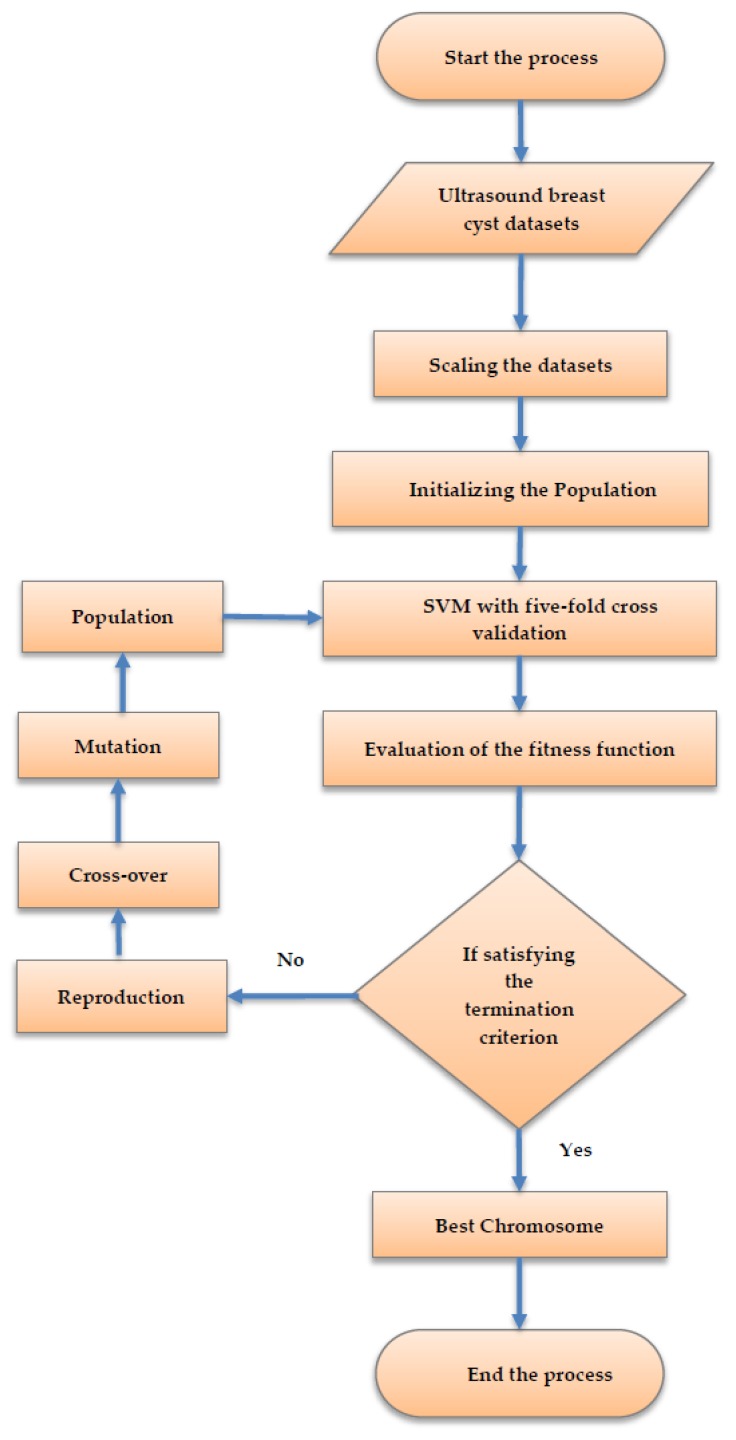
The schematic flow diagram for selecting the set of features utilizing the intelligent genetic algorithmic approach.

The schematic flow diagram for selecting the set of features utilizing the intelligent genetic algorithm is illustrated in Figure 4. Subsequently, the algorithm is discussed in the following subsection.

Stage (i): The feature values are scaled into (−1, +1) using the following equation:

(8) $$T_{i}=\frac{x_{i}-{\bar{x}}_{i}}{\sigma_{i}}$$

where $x_{i}$ signifies the value of feature *i*, ${\bar{x}}_{i}$ illustrates the mean value of $x_{i}$, $\sigma_{i}$ symbolizes the standard deviation of feature *i*. Moreover, the scaling process is performed in order to ignore the attributes in the numeric range superseding the attributes in a smaller numeric range. In addition, the accuracy rate of the support vector machine classification can also be improved utilizing this scaling of the ultrasound breast cyst images. 

Stage (ii): The process of initializing the population begins along with the chromosome encoding. These populations are generated in a random manner. Moreover, it has to be noted that all chromosomes get themselves encoded in the form of a binary string. 

Stage (iii): For all the chromosomes, the accuracy rate of the support vector machine classification is computed utilizing the five-fold cross-validation approach.

Stage (iv): The fitness function is deployed to assess all the chromosomes in the ultrasound breast cyst dataset with the aid of computing the accuracy rate of the SVM classification 

Stage (v): The process terminates itself when the stopping criterion gets fulfilled. In addition, this process gets terminated when the chromosome generation attains the value 500. During this stage, if the algorithm is still running, then this process proceeds to stage (vi).

Stage (vi): The process searches for superior solutions employing reproduction, crossover, and mutation. The stage (ii)–stage (vi) is repeated until this algorithm attains the stopping criterion.

#### 2.3.3. Support Vector Machine Approach

Moreover, this research devises the fitness function that relies on the accuracy rate of the support vector machine classification. Further, the objective of the SVM is about searching the optimal hyper-plane that segregates the ultrasound breast cyst dataset into two different classes. The support vector machine hyper-plane can be expressed using the following equation:

(9) $$f\left(x\right)=\mathrm{sgn}\left(\sum_{i=1}^{N}\alpha_{i}d_{i}K\left(x_{i},x\right)+b\right)$$

where $d_{i}$ signifies its respective class membership, the factor $x_{i}$ portrays the support vectors that are feature values of the ultrasound breast cyst images, *N* represents the total number of support vectors, and the factor $K\left(x_{i},x\right)$ denotes the kernel function. In addition, it has to note that the kernel function deployed in this research is the radial basis function (RBF) and it can be expressed using the following equation:

(10) $$K\left(x_{i},x\right)=\exp\left(-\frac{{\left\|x-x_{i}\right\|}^{2}}{2\sigma^{2}}\right)$$

where $\sigma^{2}$ specifies the kernel width. 

## 3. Results and Discussion

**Figure 5:**
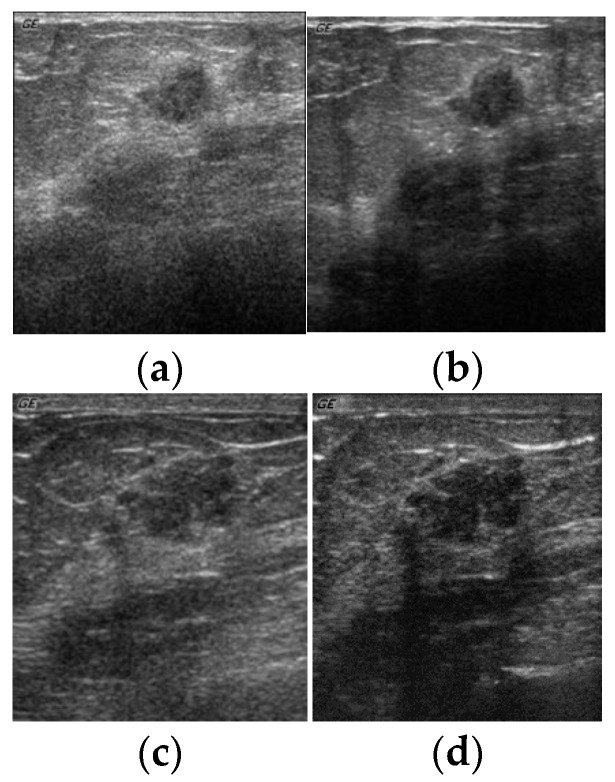
Ultrasound breast cyst image for four distinct settings of the parameters acquired from the same ultrasound sensing system (GELOGIQ 700). Breast cyst images acquired from (**a**) Parameter Setting-1, (**b**) Parameter Setting-2, (**c**) Parameter Setting-3, and (**d**) Parameter Setting-4.

All the experiments conducted for this research made use of the ultrasound breast cyst imagery dataset obtained from 23 different patients at the Department of Medical Imaging, Buddhist Dalin Tzu Chi General Hospital. Further, these images were acquired from the patients utilizing all the four distinct settings of the parameters in the ultrasound sensing system. The four distinct settings of these parameters are illustrated using Table 11. Figure 5 depicts the ultrasound breast cyst images for four distinct settings of the parameters acquired from the same ultrasound sensing system (GE GELOGIQ 700).

In general, the proposed SVM-enabled intelligent genetic algorithmic model categorizes the ultrasound breast cyst imagery datasets into two distinct groups, namely: Class 1—Invasive ductal carcinoma and Class 2—Breast Mice. Moreover, in this research, the ultrasound breast cyst imagery’s pathological information was determined with the support from an expert radiologist and was also validated using the biopsy report. The sets of the feature were extracted using the 23 × 23 mask that was applied to the ROI portion of the ultrasound breast cyst imagery. Further, overall, 4604 samples were gathered comprising of 2012 Class 1—Invasive ductal carcinoma and 2026 Class 2—Fibroadenoma samples. Among these samples, 50% were utilized for selecting the features and the rest for testing purposes.

### 3.1. Classification Results of the SVM-Enabled Intelligent Genetic Algorithmic Model-Universal Features 

In the experiments, the two-point cross over approach was deployed, and this approach had the probability and the rate of mutation as 0.65 and 0.03, respectively. Then, the possibly effective recombination solutions were selected utilizing the roulette wheel selection technique. The size of the population was chosen to be 500, and the generation process gets iterative until it reaches a value of 500. The accuracy rate of the SVM-enabled intelligent genetic algorithmic model for diverse sets of features under four distinct settings of the parameters is demonstrated in Table 12, Table 13 and Table 14, respectively. It can be witnessed from these tables that the maximum, minimum, and maximum mean accuracy values are 0.9914, 0.9581, and 0.9798, correspondingly. The value of the accuracy’s standard deviation in the case of four distinct settings of the parameters is set as 1.35, during the process where the SVM-enabled intelligent genetic algorithmic model chose the 15 sets of features. Moreover, the experimental outcomes illustrate the fact that the SVM-enabled intelligent genetic algorithmic model exhibits superior performance by selecting a highly distinguishable and essential set of features, which also accomplishes the supreme accuracy rate of classification under four distinct settings of the parameters.

### 3.2. Classification Accuracy of SVM-Enabled Intelligent Genetic Algorithmic Model for Features Chosen in Each (Individual) Parameter Setting

Generally, the chosen universal set of features for four distinct settings of the parameters can be compared in terms of the classification accuracy rate with the best feature sets chosen for each (individual) parameter settings. Further, the SVM-enabled intelligent genetic algorithmic model gets deployed for selecting the appropriate best feature sets in each (individual) parameter settings. The accuracy rate of the SVM-enabled intelligent genetic algorithmic model, when it chooses 5, 10, and 15 best feature sets under each (individual) parameter setting are represented in Table 15, Table 16 and Table 17, respectively. The mean value of the accuracy rate for classification, while choosing 5, 10, and 15 sets of features under the each (individual) parameter setting is 0.977, 0.9839, 0.9822, correspondingly. It can be witnessed from Table 12, Table 13, Table 14, Table 15, Table 16 and Table 17 that the best feature sets obtained from each (individual) parameter setting exhibit superior classification accuracy when compared with the accuracy rates of the universal set of features. The difference in the mean accuracy value amongst the universal set of features and the individual best feature sets is 1.03%, 0.57%, correspondingly. From these quantitative outcomes, it can be seen that the SVM-enabled intelligent genetic algorithmic model is supremely efficient in the selection of the universal set of features under four distinct settings of the parameters.

### 3.3. Comparison with Peer Approaches

This subsection compares the performances of the SVM-enabled intelligent genetic algorithmic model with Chang’s approach [31] and Xie’s model [32]. The performance of the SVM-enabled intelligent genetic algorithmic model and the other approaches are listed in Table 18. The mean accuracy of these methods is 97.82%, 97.58%, and 95.24%, correspondingly. The standard deviation amongst all parameter settings of these methods is 1.36, 1.93, and 2.06, correspondingly. It can be witnessed that the SVM-enabled intelligent genetic algorithmic model achieves maximum accuracy rate for classification in comparison with its peer approaches. In addition, the SVM-enabled intelligent genetic algorithmic model also exhibits a low value for the standard deviation metric when compared with other approaches.

### 3.4. Discussion 

In Table 12, the classification results of the proposed SVM-enabled intelligent genetic algorithmic model chooses five universal features is illustrated with accuracy rate 0.9842 (Parameter Setting-1), 0.9681 Parameter Setting-2), 0.9752 (Parameter Setting-3), and 0.9427 (Parameter Setting-4), respectively. Moreover, in Table 13, the classification results of the proposed method chooses 10 universal features illustrated with accuracy rates 0.9835 (Parameter Setting-1), 0.9886 (Parameter Setting-2), 0.9827 (Parameter Setting-3), and 0.9581 (Parameter Setting-4), respectively. In addition, in Table 14, the classification results of the proposed method chooses 15 universal features is illustrated with accuracy rates 0.9914 (Parameter Setting-1), 0.9848 (Parameter Setting-2), 0.9827 (Parameter Setting-3), and 0.9602 (Parameter Setting-4), respectively. Hence, it can be witnessed from Table 12, Table 13 and Table 14 that when there is an increase in the number of the universal features (5, 10, and 15), there is a corresponding increase in the accuracy rate and the mean accuracy rate. 

Moreover, in Table 15, the classification results of the proposed method choose five features in each parameter setting is portrayed with accuracy rate 0.99 (Parameter Setting-1), 0.9717 (Parameter Setting-2), 0.9804 (Parameter Setting-3), and 0.9691 (Parameter Setting-4), respectively. In addition, in table 16, the classification results of the proposed method chooses ten features in each parameter setting is portrayed with accuracy rate 0.9934 (Parameter Setting-1), 0.9894 (Parameter Setting-2), 0.9842 (Parameter Setting-3), and 0.9686 (Parameter Setting-4), respectively. Subsequently, in Table 17, the classification results of the proposed method choose 15 features in each parameter setting is portrayed with accuracy rate 0.9927 (Parameter Setting-1), 0.9894 (Parameter Setting-2), 0.9838 (Parameter Setting-3), and 0.9631 (Parameter Setting-4), respectively. Therefore, it can be witnessed from Table 15 and Table 16 that when there is an increase in the number of the individual features from 5 to 10, there is a corresponding increase in the accuracy rate and the mean accuracy rate. However, in general, there is a drop in both the accuracy rate and the mean accuracy rate when there is an increase in the number of individual features from 10 to 15. The experimental results exhibit the fact that the proposed SVM-enabled intelligent genetic algorithmic model possesses significant potential in the selection of appropriate best feature sets, which can assist in the early detection of breast cancer.

## 4. Conclusions

The cause of death among Taiwanese and worldwide women due to malignant breast cancer is widely increasing in the present day scenario. Moreover, the ultrasound sensing systems perform well in the diagnosis of the lesion region, and it can clearly distinguish it from the healthy tissues in the breast. Moreover, it has to be noted that the resolution achieved by these ultrasound sensing systems is not so high. Consequently, this makes the task of classifying the breast cysts arduous for medical doctors through direct vision. Furthermore, obtaining the breast cyst imagery of a patient through four distinct settings of the parameters in an ultrasound sensing system might be useful in avoiding the invasive procedures. Therefore, this research establishes the SVM-enabled intelligent genetic algorithmic model for selecting the universal set of features that can easily aid in distinguishing between the breasts with and without cysts. Further, the evaluation metrics such as Sorensen-Dice coefficient, *t*-test, and Pearson’s R measure is utilized for assessing the extracted feature sets. The SVM-enabled intelligent genetic algorithmic model is deployed for choosing a highly discriminant and relevant set of features. The experimental outcomes indicate that the SVM-enabled intelligent genetic algorithmic model accomplishes a superior accuracy rate for classification under four distinct settings of the parameters. Moreover, the SVM-enabled intelligent genetic algorithmic model surpasses the other compared peer-level approaches in terms of classification accuracy; also, it converges (generalizes) well in the case of distinct settings of the parameters. In addition, the proposed model also enables medical doctors in the detection of fibroadenoma and invasive ductal carcinoma (IDC). Moreover, an advanced ultrasound system with real-time autonomous diagnosis capabilities can be considered as the task for the future. With sufficiently large ultrasound image datasets, deep learning models can be utilized in the future for efficient disease diagnosis. 

## Figures and Tables

**Table 1:** Causes of cancer death, 2015 and 2016 (Unit: Persons, %).

ICD-10 Mortality NO.	Causes of Cancer Death	2016					2015				
		Rank	Number of Deaths	% of All Deaths	Per 100,000 Population		Rank	Number of Deaths	% of All Deaths	Per 100,000 Population	
					Crude Death Rate	Age-Adjusted Death Rate				Crude Death Rate	Age-Adjusted Death Rate
C00–C97	Malignant neoplasms		47,760	100.0	203.1	126.8		46,829	100.0	199.6	128.0
C33–C34	Cancers of trachea, bronchus and lung	1	9372	19.6	39.9	24.4	1	9232	19.7	39.3	24.7
C22	Cancers of liver and intrahepatic bile ducts	2	8353	17.5	35.5	22.2	2	8258	17.6	35.2	22.8
C18–C21	Cancers of colon, rectum and anus	3	5722	12.0	24.3	14.6	3	5687	12.1	24.2	14.9
C50	Cancer of breast (Female)	4	2176	4.6	18.4	11.8	4	2141	4.6	18.2	12.0
C00–C06, C09–C10, C12–C14	Cancer of oral cavity	5	2936	6.1	12.5	8.3	5	2667	5.7	11.4	7.8
C61	Cancer of prostate	6	1347	2.8	11.5	6.8	6	1231	2.6	10.5	6.4
C16	Cancer of stomach	7	2315	4.8	9.8	5.8	7	2326	5.0	9.9	6.1
C25	Cancer of pancreas	8	1996	4.2	8.5	5.3	8	1948	4.2	8.3	5.3
C15	Cancer of oesophagus	9	1731	3.6	7.4	4.8	9	1807	3.9	7.7	5.1
C56, C57.0–C57.4	Cancer of ovary	10	656	1.4	5.6	3.6	12	529	1.1	4.5	3.0

**Table 2:** Histogram feature sets.

Feature Name	Feature Number
Energy	F1
Entropy	F2
Kurtosis	F3
Mean	F4
Skewness	F5
Standard Deviation	F6
Variance	F7

**Table 3:** Gray-level spatial dependence matrix feature sets.

Feature Name	Feature Number
Correlation	F8
Difference of entropy	F9
Difference of variance	F10
Sum of average	F11
Sum of entropy	F12
Sum of squares	F13
Sum of variance	F14
Contrast	F15
Energy	F16
Entropy	F17
Local homogeneity	F18
Cluster shade	F19
Cluster prominence	F20

**Table 4:** Statistical feature matrix feature.

Feature Name	Feature Number
Dissimilarity	F21

**Table 5:** Gray level run-length textural matrix feature sets.

Feature Name	Feature Number
Short-run emphasis	F22
Long-run emphasis	F23
Gray-level uniformity	F24
Run-length uniformity	F25
Run percentage	F26

**Table 6:** Laws’ texture energy matrix feature sets.

Feature Name	Feature Number
LE mean	F27
EL mean	F28
SL mean	F29
EE mean	F30
LS mean	F31
LE variance	F32
EL variance	F33
SL variance	F34
EE variance	F35
LS variance	F36

**Table 7:** Neighboring gray level dependence matrix feature sets.

Feature Name	Feature Number
Small number emphasis	F37
Large number emphasis	F38
Number non-uniformity	F39
Second moment	F40
Entropy	F41

**Table 8:** Neighborhood gray tone difference matrix feature sets.

Feature Name	Feature Number
Busyness	F42
Coarseness	F43
Complexity	F44
Contrast	F45
Textural Strength	F46

**Table 9:** Wavelet transform feature sets.

Feature Name	Feature Number(s)
Histogram features	F47–F53
GLSDM features	F54–F66
SFM feature	F67
GLRLM features	F68–F72
Mean	F73
Standard deviation	F74
Laws’ features	F75–F84
NGLDM features	F85–F89
NGTDM features	F90–F94

**Table 10:** Fourier features—local Fourier coefficients.

Feature Name	Feature Number(s)
Means of eight magnitudes	F95–F102
Means of eight phase angles	F103–F110
Standard deviations of the eight magnitudes	F111–F118
Standard deviations of the eight phase angles	F119–F126

**Table 11:** The four distinct settings of the parameters in the ultrasound sensing system (GELOGIQ 700).

Parameter Setting Number	Details
GELOGIQ700-Parameter Setting-1	The probe is a B-mode linear array. The depth range = 4 cm; dynamic range = 66 dB; gain = 36; edge enhance = E3; gray map = MD; frame average setting = A3.
GELOGIQ700-Parameter Setting-2	The probe is a B-mode linear array. The depth range = 4 cm; dynamic range = 69 dB; gain = 35; edge enhance = E2; gray map = MC; frame average setting = A3.
GELOGIQ700-Parameter Setting-3	The probe is a B-mode linear array. The depth range = 3 cm; dynamic range = 69 dB; gain = 34; edge enhance = E3; gray map = MC; frame average setting = A3.
GELOGIQ700-Parameter Setting-4	The probe is a B-mode linear array. The depth range = 3 cm; dynamic range = 63 dB; gain = 34; edge enhance = E2; gray map = MD; frame average setting = A4.

**Table 12:** Classification results of the SVM-enabled intelligent genetic algorithmic model when it chooses five universal features.

Ultrasound Sensing System Setting Detail	Accuracy Rate (%)
Parameter Setting-1	98.42
Parameter Setting-2	96.81
Parameter Setting-3	97.52
Parameter Setting-4	94.27
Mean	96.75

**Table 13:** Classification results of the SVM-enabled intelligent genetic algorithmic model when it chooses 10 universal features.

Ultrasound Sensing System Setting Detail	Accuracy Rate (%)
Parameter Setting-1	98.35
Parameter Setting-2	98.86
Parameter Setting-3	98.27
Parameter Setting-4	95.81
Mean	97.82
Feature sets	F4, F5, F22, F26, F30, F95, F108, F109, F113, F124

**Table 14:** Classification results of the SVM-enabled intelligent genetic algorithmic model when it chooses 15 universal features.

Ultrasound Sensing System Setting Detail	Accuracy Rate (%)
Parameter Setting-1	99.14
Parameter Setting-2	98.48
Parameter Setting-3	98.27
Parameter Setting-4	96.02
Mean	97.98
Feature sets	F4, F5, F14, F22, F25, F29, F77, F103, F106, F108, F109, F113, F115, F121, F124

**Table 15:** Classification results of the SVM-enabled intelligent genetic algorithmic model when it chooses five features in each parameter setting.

Ultrasound Sensing System Setting Detail	The Best Feature Sets	Accuracy Rate (%)
Parameter Setting-1	F4, F5, F105, F121, F11	99.00
Parameter Setting-2	F4, F29, F37, F19, F1	97.17
Parameter Setting-3	F39, F107, F6, F37, F1	98.04
Parameter Setting-4	F4, F15, F51, F47, F124	96.91

**Table 16:** Classification results of the SVM-enabled intelligent genetic algorithmic model when it chooses 10 features in each parameter setting.

Ultrasound Sensing System Setting Detail	The Best Feature Sets	Accuracy Rate (%)
Parameter Setting-1	F4, F5, F105, F3, F11, F40, F6, F1, F2, F7	99.34
Parameter Setting-2	F4, F29, F9, F22, F1, F12, F8, F37, F5, F32	98.94
Parameter Setting-3	F4, F107, F5, F37, F1, F2, F3, F6, F113, F9	98.42
Parameter Setting-4	F4, F15, F5, F17, F123, F109, F1, F104, F106, F2	96.86
Mean		98.39

**Table 17:** Classification results of the SVM-enabled intelligent genetic algorithmic model when it chooses 15 features in each parameter setting.

Ultrasound Sensing System Setting Detail	The Best Feature Sets	Accuracy Rate (%)
Parameter Setting-1	F4, F5, F105, F3, F11, F40, F6, F1, F2, F7, F10, F13, F9, F12, F8	99.27
Parameter Setting-2	F4, F29, F9, F22, F1, F12, F8, F37, F5, F32, F48, F2, F6, F10, F7	98.94
Parameter Setting-3	F4, F107, F5, F37, F1, F2, F3, F6, F113, F9, F10, F15, F11, F21, F16	98.38
Parameter Setting-4	F4, F15, F5, F17, F123, F109, F1, F104, F106, F2, F3, F6, F7, F9, F11	96.31
Mean		98.22

**Table 18:** Classification results of SVM-enabled intelligent genetic algorithmic model and peer approaches when chooses 10 features in each parameter setting.

Ultrasound Sensing System Setting Detail	The SVM-Enabled Intelligent Genetic Algorithmic Model	Chang’s Approach [31]	Xie’s Model [32]
Parameter Setting-1	98.35%	99.80%	95.87%
Parameter Setting-2	98.86%	97.79%	96.23%
Parameter Setting-3	98.27%	97.76%	96.72%
Parameter Setting-4	95.81%	95.09%	92.12%
Mean	97.82%	97.58%	95.24%
The best feature sets	F4, F5, F26, F22, F30, F95, F108, F109, F113, F124	F4, F112, F44, F99, F122, F78, F11, F120, F5, F22	F6, F101, F98, F44, F56, F2, F104, F31, F113, F108
